# A systematic review and meta-analysis of haematological malignancies in residents living near petrochemical facilities

**DOI:** 10.1186/s12940-020-00582-1

**Published:** 2020-05-19

**Authors:** Calvin Jephcote, David Brown, Thomas Verbeek, Alice Mah

**Affiliations:** 1 Department of Sociology, University of Warwick, Coventry, CV4 7AL UK; 2 Centre for Environmental Health and Sustainability, University of Leicester, Leicester, LE1 7HA UK

**Keywords:** Cancer, Environmental justice, Haematological, Leukaemia, Lymphoma, Myeloma, Meta-analysis, Petrochemical, Refinery

## Abstract

**Background:**

The petrochemical industry is a major source of hazardous and toxic air pollutants that are recognised to have mutagenic and carcinogenic properties. A wealth of occupational epidemiology literature exists around the petrochemical industry, with adverse haematological effects identified in employees exposed to ‘low’ concentrations of aromatic hydrocarbons (benzene, toluene, ethylbenzene, and xylene). Releases from the petrochemical industry are also thought to increase the risk of cancer incidence in fenceline communities. However, this emerging and at times inconclusive evidence base remains fragmented. The present study’s aim was to conduct a systematic review and meta-analysis of epidemiological studies investigating the association between incidences of haematological malignancy and residential exposure to the petrochemical industry.

**Methods:**

Epidemiological studies reporting the risk of haematological malignancies (Leukaemia, Hodgkin’s lymphoma, Non-Hodgkin’s lymphoma, and Multiple myeloma) were included where the following criteria were met: (i) Cancer incidence is diagnosed by a medical professional and coded in accordance to the International Classification of Diseases; (ii) A clear definition of fenceline communities is provided, indicating the proximity between exposed residents and petrochemical activities; and (iii) Exposure is representative of normal operating conditions, not emergency events. Two investigators independently extracted information on study characteristics and outcomes in accordance with PRISMA and MOOSE guidelines. Relative risks and their 95% confidence intervals were pooled across studies for the four categories of haematological malignancy, using a random effects meta-analysis.

**Results:**

The systematic review identified 16 unique studies, which collectively record the incidence of haematological malignancies across 187,585 residents living close to a petrochemical operation. Residents from fenceline communities, less than 5 km from a petrochemical facility (refinery or manufacturer of commercial chemicals), had a 30% higher risk of developing Leukaemia than residents from communities with no petrochemical activity. Meanwhile, the association between exposure and rarer forms of haematological malignancy remains uncertain, with further research required.

**Conclusions:**

The risk of developing Leukaemia appears higher in individuals living near a petrochemical facility. This highlights the need for further policy to regulate the release of carcinogens by industry.

**Graphical abstract:**

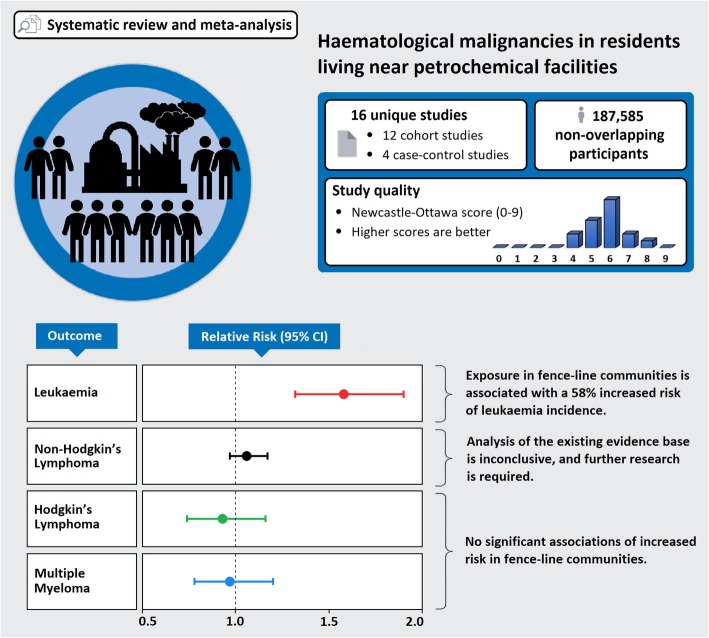

## Introduction

Haematological malignancies are cancers affecting the blood, bone marrow, lymph, and lymphatic system. The International Agency for Research on Cancer (IARC) classifies haematological malignancies into four broad categories: Leukaemia, Hodgkin Lymphoma, Non-Hodgkin Lymphoma and Multiple Myeloma [1, 2]. In line with global cancer trends, estimated incidences and mortality of blood cancers are rapidly rising, with the number of new leukaemia cases estimated at 437,033 and the number of deaths attributable to leukaemia estimated at 309,006 in 2018, according to the Global Cancer Statistics (see Table 1). Several agents contribute to the development of blood cancers, including occupational, lifestyle, and hereditary risk factors [3]. People may be exposed to carcinogens in their lived environments, notably through chronic and acute forms of air pollution [4, 5].

**Table 1:** The different haematological malignancies

Condition	Definition [2]	Global incidence per annum [4]		Global mortalities per annum [4]	
		Count	ASR ^**a**^	Count	ASR ^**a**^
Leukaemia	A type of cancer that develops in blood-forming tissue, normally the bone marrow, resulting in the production of abnormal white blood cells which crowd out normal blood cells and platelets.	437,033	5.2	309,006	3.5
Hodgkin’s Lymphoma	A cancer of the lymphatic system, a large network of nodes and vessels that carries tissue fluid (called lymph) throughout the body and an important element of the immune system.	79,990	1	26,167	0.3
Non-Hodgkin’s Lymphoma	A cancer of the lymphatic system.	509,590	5.7	248,724	2.8
Multiple Myeloma	A cancer of plasma cells, a type of white blood cells that produce antibodies, which collects in the bone marrow, the soft fatty tissue inside bone cavities.	159,985	1.8	106,105	1.1

^**a**^ Age-standardized rates (ASRs) per 100,000 persons

The petrochemical industry acts as a major source of hazardous and toxic air pollution and is associated with the release of a range of known carcinogens, such as volatile organic compounds, BTEX (benzene, toluene, ethylbenzene, xylene), polycyclic aromatic hydrocarbons, polychlorinated biphenyls and polyvinyl chloride [6, 7]. The petrochemical industry incorporates the ‘upstream’ processing of crude oil and natural gas, the ‘midstream’ storage and transportation of refined oil and gas products, and the ‘downstream’ manufacturing of petrochemicals and commercially marketable products. As a way of reducing transportation costs and mitigating safety concerns, and due to agglomeration economies and integrated production processes, several refinery and manufacturing operations are often clustered in petrochemical industrial complexes, increasing combined pollutant levels [8]. Given that petrochemicals make up part of numerous everyday commodities, it is often difficult to distinguish where the petrochemical industry begins and ends.

A wealth of occupational studies have identified adverse haematological effects in employees exposed to toxicants in the petrochemical industry [9]. Notably, occupational benzene exposure has been found to increase the risk of haematological malignancies among workers, even with low recorded daily concentrations (< 0.1 ppm) [10–14]. The increased risk has been consistently demonstrated through identification of DNA or chromosomal damage [15–17], reduced white blood cell counts [18, 19], and case control studies [20–22]. The raised occupational risk of developing haematological malignancies due to occupational benzene exposure has been observed across the upstream, midstream and downstream sectors of the petrochemical industry, notably incorporating studies of refineries [23–26], synthetic rubber manufacturing plants [27–30], petrochemical [31–35], and plastics manufacturing industries [21, 36], as well as petroleum storage and distribution [37]. Accordingly, the World Health Organisation (WHO) [38] states that there are no safe levels of benzene exposure, associating it with an excess lifetime risk of leukaemia.

While evidence on the elevated occupational risk of developing haematological malignancies in the petrochemical industry is largely conclusive, toxic and hazardous releases from the industry are also understood to increase the risk of cancer incidence in fenceline communities. However, the evidence base documenting the blood cancer risks for populations living in close proximity to petrochemical operations remains inconclusive and disjointed. There is a need to enhance understanding of the specific health impacts of the petrochemical industry for fenceline communities, as an evidence base for environmental (in)justices.

To our knowledge, there has been no meta-analysis thus far on the incidence of haematological malignancies in fenceline communities living near petrochemical sites. To fill this gap, we conduct a meta-analysis of cohort and case-control studies that have examined the incidences of haematological malignancies in residential populations close to petrochemical sites. For this we build upon Lin et al.’s meta-analyses of lung cancer mortality [8] and incidence [7] in fenceline communities residing close to petrochemical industrial complexes.

## Methods

### Search strategy

**Fig. 1:**
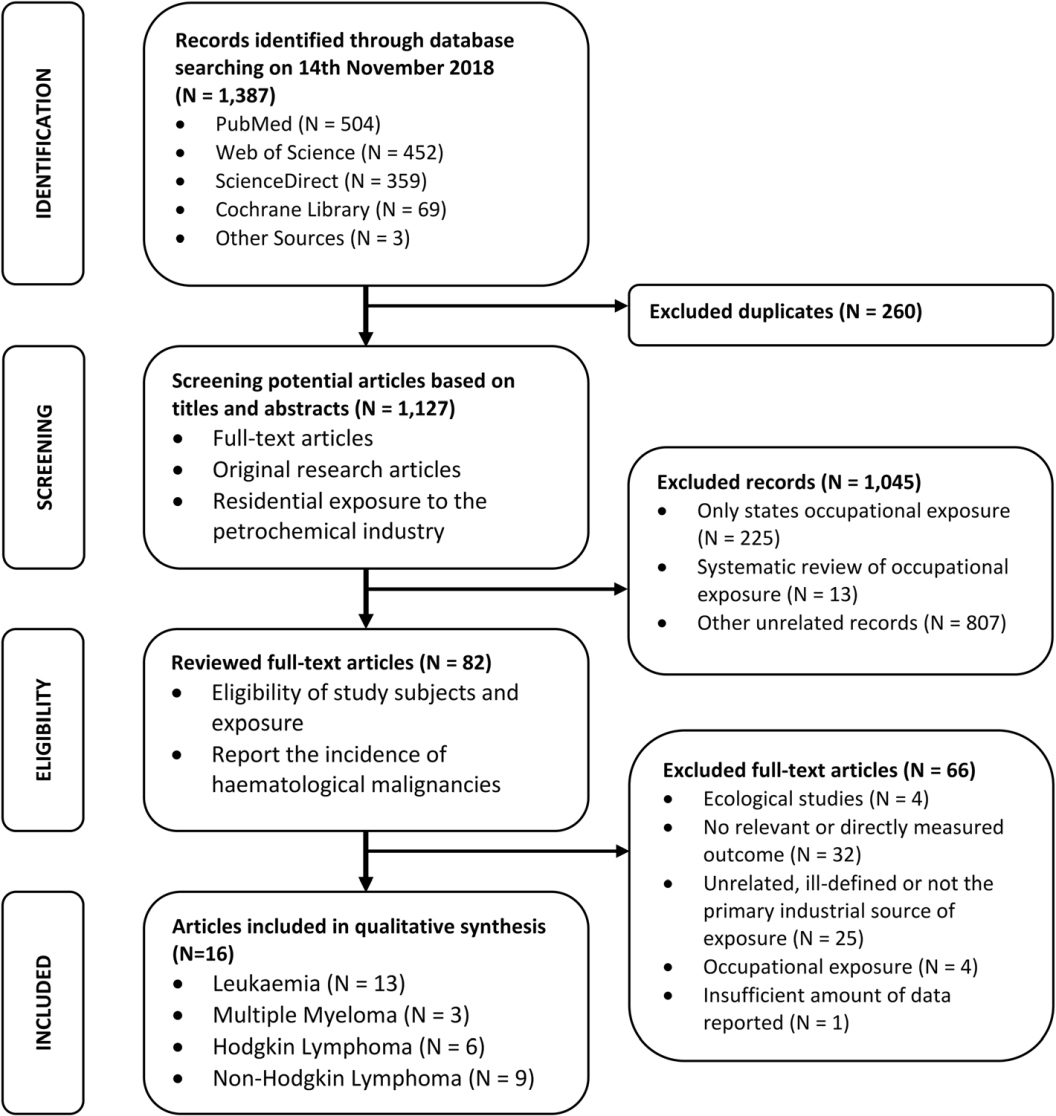
PRISMA flowchart of the systematic literature search on haematological malignancy incidence in residents living near petrochemical facilities

The study was conducted in accordance to PRISMA and MOOSE guidelines (see Fig. 1 and the checklists contained in Additional file 1). The meta-analysis protocol was registered in the PROSPERO database (ID: CRD42019118567), to avoid the duplication of research and for transparency purposes by providing evidence of a priori analysis intentions. This procedure minimises the introduction of bias by hypothesising after the results are known, otherwise known as ‘HARRKing’, whereby findings are omitted or accommodated to achieve a more desirable outcome [39].

We comprehensively searched the Cochrane Library, PubMed, ScienceDirect and Web of Science electronic databases for published studies available before Wednesday 14th November 2018. The following search terms were used to look for partial matches in the abstract, title, and keywords: (“Haematological Malignancy” OR “Blood Cancer” OR “Leukaemia” OR “Lymphoma” OR “Myeloma” OR “Hodgkin”) AND (“Refinery” OR “Petroleum” OR “Petrochemical” OR “Oil Industry” OR “Gas Industry” OR “Chemical Industry”). The search criteria were set to include full-text publications from any year and in all languages, with wildcards and spelling variations between the American and British English language accounted for (see Additional file 1). Only a few articles of relevance from the returned literature were published in languages other than English (Italian), but these were excluded for reporting rates of mortality rather than incidence. Additional studies were obtained through manually searching the reference lists of articles that were identified to be of importance. The returned search results were collated and processed using EndNote 19.0.

### Selection criteria

Epidemiological studies reporting the risk of haematological malignancies (Leukaemia, Hodgkin’s lymphoma, Non-Hodgkin’s lymphoma, or Multiple myeloma) were included where the following criteria were met: (i) Cancer incidence is diagnosed by a medical professional and coded in accordance to the International Classification of Diseases; (ii) A clear definition of fenceline communities is provided, indicating the proximity between exposed residents and petrochemical activities; and (iii) Exposure is representative of normal operating conditions, not emergency events.

Ecological studies comparing incidence rates in regions with and without petrochemical activity were excluded, based on issues of representativeness. The proximity of individuals to petrochemical sources and therefore the proportion of the population exposed in such studies is unknown, with any group-level measures of confounding influences not necessarily providing a true representation of individual experiences. However, this ecological literature contains the only record of haematological malignancy rates for the oil fields of Croatia [40] and the Ecuadorian Amazon basin [41, 42], the exclusion of which will restrict the geographic coverage of our analysis.

Other noteworthy exclusions include:

- Knox’s (1994) [43] exploratory case-control study of leukaemia in British children aggregated cases into postal zones and randomly assigned a census-based control cluster, using potentially unrelated socio-economic characteristics and an unknown level of exposure to downstream petrochemical activity.
- Patel’s (2004) [44] cohort study of a large spill event at an underground storage facility in Pennsylvania was also excluded, because exposure was not representative of normal midstream operating conditions.

Two reviewers independently screened the search results for conformity with the selection criteria, and any disagreement was resolved by a third reviewer.

### Data extraction and quality assessment

Two investigators used standardised protocols to independently extract descriptive data for the following characteristics: study design, study location (country and continent), study resolution (temporal and geographic), exposure by petrochemical sector (upstream, downstream or midstream), community proximity to the petrochemical industry, year of the baseline survey, age range of participants at the baseline, gender, diagnostic classification system (ICD 7–10 and ICD-O), and the degree of statistical adjustment used to account for confounding influences. The following procedure was conducted to collect missing information: (1) contact the articles corresponding authors; (2) If an inactive account exists, academic profiles were explored using the academic databases mentioned in section 2.1 and internet search engines; (3) Records were estimated from census records and other registers, if no response was received to the first two stages (see Table 3).

Two reviewers independently used the Newcastle-Ottawa scale to assess the quality and potential risk of bias in the included studies, with any disagreement resolved by a third reviewer [45]. This 10-point scale (0–9) provides a semi-quantitative evaluation of a study’s selection of participants, comparability, and outcomes. See Additional file 1 for examples of the quality scoring criteria.

A single effect size was extracted from each primary study, except when the authors had provided gender or source specific (i.e. nonadjacent communities exposed to a different form of petrochemical activity) approximations of relative risk. This approach allowed for the investigation of moderator effects that are of interest, while minimising the risk of dependency between effect sizes. Often effect sizes from the same study or research group are more alike and thus interdependent, because of similarities in study design, measurement, analysis, and the selection of participants – influences, which if ignored can inflate and lead to the overconfidence of a meta-analysis [46, 47]. This approach contrasts from that of Lin et al. [7], the only other meta-analysis of incidence rates in residents near to petrochemical facilities. Lin et al’s [7] analysis of lung cancer incidence included 17 approximations of relative risk taken from 6 studies, with ten risk measures coming from a single study of Sicily [48] providing 71.7% of the weight behind the pooled estimate. A conservative approach would have only extracted male and female risk estimates for the entire contamination zone, instead of including separate results for each individual municipality. Lin et al’s [7] meta-analysis also fails to provide an adequate definition of fenceline communities, either by proximity or pollution thresholds, resulting in the inclusion of the ecological study by Hurtig & San Sebastián [41].

In our analysis, Fazzo et al.’s [48] study only provides gender specific haematological incidence rates for Priolo, because no other municipalities in the contamination zone are within 5 km of a petrochemical facility.

### Data synthesis

Estimates from the individual studies were reported either as an Odds Ratio (*n* = 4) or Standardised Incidence Ratio (*n* = 12). The Odds Ratio (OR) and Standardised Incidence Ratio (SIR) may be considered as approximations of Relative Risk (RR) under the rare disease assumption, where the rate of such an event in the general population is less than 10% [8, 49–51].

These approximations of RR were log-transformed, to ensure the distribution of these outcome measure are symmetric around 0 and are close to normal [52]. Twelve studies provided upper and lower 95% confidence interval limits, which were converted into Standard Error (SE) estimates of the natural-log RR with the following equation [53]:

$$ \mathbf{SE}\left(\mathbf{lnRR}\right)=\frac{\mathbf{\ln}\left({\mathbf{RR}}_{\mathbf{upper}}\right)-\mathbf{\ln}\left({\mathbf{RR}}_{\mathbf{lower}}\right)}{\mathbf{2}\times \mathbf{1.96}} $$

Where **ln**(**RR**_**upper**_) and **ln**(**RR**_**lower**_) represent the natural logs of the upper limit and lower limit of RR, respectively.

Four studies [54–57] only provide information on the observed (**O**) number of incident cases in the exposed group and the expected (**E**) number of cases based on a reference population, to calculate the SIR where **O/E**. The 95% confidence intervals of these studies were estimated by regarding **O** as a Poisson variable, with upper (**O**_**U**_) and lower (**O**_**L**_) distribution table values that are to be divided by **E** [58]. These confidence intervals were then converted into SE estimates of the natural-log RR.

### Statistical analysis

Relative risks and their 95% confidence intervals were pooled across studies for each of the four categories of haematological malignancy, using a random effects meta-analysis. We assessed the consistency of findings across individual studies with the I^2^ test, where a score of less than 25% indicates a low level of between-study heterogeneity [59]. A higher test score would indicate that variation in the effect estimates is not a result of chance, but of the presence of a moderator effect, that has in part influenced the direction and strength of the study outcome. Meta-regressions containing a single quantitative or qualitative moderator effect were used to investigate possible causes of heterogeneity and when required to construct adjusted risk ratios, based on demographic, diagnosis, exposure, geographic, quality or temporal differences in the study designs.

Contour-enhanced funnel plots [60] collectively displayed the study effect estimates against their standard errors, to check for the presence of publication bias, whereby inconclusive results are more likely to remain unpublished. Funnel lines were centred at 0 (i.e. the null hypothesis of no effect), with the observation effects classified by confidence interval bands. The visual presence of asymmetry is a subjective indicator of publication bias. Egger’s regression test was then employed to objectively determine if the effect estimates and sampling variances are related, with *p*-values < 0.05 indicative of publication bias [61].

It should be noted that the funnel plot and its associated measures are only capable of testing for ‘positive’ forms of publication bias, and it is plausible that research on industrial pollution and public health, may in part be suppressed, if the publication of positive associations had the potential to cause economic damage. As meta-analysis guidelines do not currently account for ‘negative’ forms of publication bias, we can only place a limited amount of influence on such tests, which were conducted in accordance to PRISMA and MOOSE guidelines.

All components of this analysis were conducted in the open source [R] programming language, using the ‘metafor’ package version 2.1 [52].

### Case-study: Louisiana’s petrochemical corridor

Activists have long argued that the petrochemical industry has severely harmed the health of people living in Louisiana; however, first-hand quantitative evidence has been notoriously difficult to gather: Research has reportedly been terminated over safety fears, and local cancer registries have been spatiotemporally censored at low resolution units, debatably over disclosure concerns or to conveniently mask trends within the data [62, 63].

Using the knowledge gained from these meta-analysis models, we intend to conservatively estimate the impact of petrochemical activity on haematological malignancy incidence in Louisiana from 2011 to 15, based on the following approach.

While the ‘Upstream’ component of the petrochemical industry is clearly defined (i.e. refineries), difficulties exist in the identification of ‘Downstream’ activities, and links start to blur as petroleum feedstock is gradually restructured into ever more complex chemical components. Still, most forms of petrochemical activity may be traced by the release of volatile aromatic hydrocarbons, in the form of benzene, toluene, ethylbenzene, and xylene compounds (BTEX).

We assumed that ‘Upstream’ activities are the most polluting aspect of the industry, as they process and break down the largest volumes of petrochemical feedstock. Table 2 provides the median annual emission rates of BTEX compounds for all petrochemical refineries in the United States, in 1987 and for 2011–15. Environmental legislation over the past 30 years has seen a cleaning of industry; however, facilities still exist that are considered highly polluting even by historic standards (i.e. above the 1987 median BTEX values).

**Table 2:** Median annual emission rates for all petrochemical refineries in the United States. (Source: US-EPA Toxic Release Inventory 1987-2015 [64])

Year(s)	Count	BTEX Emissions (tonnes per annum)			
		Benzene	Toluene	Ethylbenzene	Xylenes
1987	144	6.19	15.88	2.37	8.94
2011–2015	145	2.90	5.14	1.00	4.24

We first identified all industries operating in Louisiana between 2011 and 2015, which released emissions of a BTEX compound above or equal to the median emission level of refineries in 1987 (*n* = 29). The selected facilities are considered as ‘highly’ polluting, even by historic levels. Standard industrial classification (SIC) codes identified 8 refineries, 1 midstream storage facility, and 10 downstream manufacturers of commercially marketable products in the form of organic chemicals, plastics and fertilisers. Fifteen of these ‘highly’ polluting petrochemical facilities were located on the Mississippi (79%). Ten facilities were omitted from the analysis for not processing hydrocarbons, which highlights the additional risk posed by other industrial activity, particularly from the fabrication of metals at shipyards.

Firstly, the total number of leukaemia cases in each parish was calculated, by combining population counts with age-adjusted incidence rates obtained from the National Cancer Institute. The US Environmental Protection Agency’s Toxic Release Inventory (TRI) was then used to identify ‘highly’ polluting petrochemical facilities, and a 30x30m population grid was used to estimate the size of their fenceline communities [65].

For each individual parish, a ‘Population Attributable Fraction’ (PAF) estimated the proportion of haematological malignancy incidence, attributable to residential exposure from the petrochemical industry [66]:

$$ \mathbf{PAF}=\frac{{\mathbf{P}}_{\mathbf{pop}}\times \left(\mathbf{RR}-\mathbf{1}\right)}{{\mathbf{P}}_{\mathbf{pop}}\times \left(\mathbf{RR}-\mathbf{1}\right)+\mathbf{1}} $$

Where **P**_**pop**_ represents the proportion of a parish residing within 5 km of a petrochemical facility (i.e. the proportion of exposed subjects), and **RR** is the pooled relative risk of a particular outcome occurring in the exposed population, as estimated by the meta-analysis models.

## Results

### Study level characteristics

The systematic review identified 16 unique studies, which collectively record the incidence of haematological malignancies across 187,585 residents living close to a petrochemical operation (see Table 3) [6, 48, 54–57, 67–76]. In terms of geographical coverage, 11 studies were conducted in Europe, 3 in North America and 2 in Asia. Twelve of these studies were based on retrospective cohorts, with the remaining four implementing a case-control approach. As a collective, the systematically identified literature covers 51 years of petrochemical activity (1960–2011).

**Table 3:** A summary of the studies identified by the systematic review for inclusion in the meta-analysis models (based on design, survey resolution and the demographics of fenceline communities)

Lead Author (Publication Year)	Country	Study Design	Level of Adjustment ^a^	Survey Resolution		Petrochemical Activity				Exposed Population ^c^						
				Temporal	Spatial	Source of Exposure (N) ^**b**^			Proximity to Source (km)	Count (N)	Mean Age (Years)	Male (%)	Cases (N) ^**d**^			
						UP	MID	DWN					LK	MM	HL	NHL
Axelsson (2010) [6]	Sweden	Cohort	Low	1994–2005	Local	–	–	4	≤ 2	2800 (b)	0 to 65+	50 (b)	10	–	–	–
Barregard (2009) [68]	Sweden	Cohort	Low	1975–2004	Local	1	–	–	≤ 5	5000	0 to 65+	50 (b)	33	–	–	–
Beale (2010) [69]	USA	Cohort	Moderate	1973–2006	Local	5	–	–	≤ 2.5	27,100 (b)	0 to 65+	100	145	52	34	184
Beale (2010) [69]	USA	Cohort	Moderate	1973–2006	Local	5	–	–	≤ 2.5	26,900 (b)	0 to 65+	0	100	35	30	141
Bulat (2011) [56]	Serbia	Cohort	Low	2003–2008	Local	1	–	2	≤ 7.5	26,375 (a)	41 (a)	100	15	–	12	21
Bulat (2011) [56]	Serbia	Cohort	Low	2003–2008	Local	1	–	2	≤ 7.5	39,828 (a)	41 (a)	0	15	–	7	14
De Roos (2010) [70]	USA	Case-Control	High	1988–2000	Subnational	15	–	33	≤ 3.2	864	58	53	–	–	–	94
Fazzo (2016) [48]	Italy	Cohort	Low	1999–2006	Local	2	–	1	≤ 3	101	0 to 65+	100	8	4	4	4
Fazzo (2016) [48]	Italy	Cohort	Low	1999–2006	Local	2	–	1	≤ 3	37	0 to 65+	0	1	4	3	5
García-Pérez (2015) [71]	Spain	Case-Control	Moderate	1990–2011	National	–	–	96	≤ 2.5	872	< 15	58	44	–	–	–
Linos (1991) [72]	USA	Case-Control	High	1980–1983	Subnational	29	–	–	≤ 3.2	33	< 65	100	15	–	–	14
Linos (1991) [72]	USA	Case-Control	High	1980–1983	Subnational	–	–	28	≤ 3.2	59	< 65	100	27	–	–	18
Lyons (1995) [54]	UK	Cohort	Low	1974–1991	Local	–	–	1	≤ 1.5	2632	< 25	53 (b)	–	–	2	–
Pasetto (2012) [73]	Italy	Cohort	Moderate	1960–2000	Local	1	–	–	≤ 5	2419	53	100	–	–	–	11
Pekkanen (1995) [55]	Finland	Cohort	Low	1983–1986	Local	1	–	–	≤ 8	1250	0 to 65+	51	2	–	–	–
Salerno (2013) [74]	Italy	Cohort	Low	2003–2009	Local	1	–	–	≤ 3	3360 (b)	0 to 65+	100	6	2	1	10
Salerno (2013) [74]	Italy	Cohort	Low	2003–2009	Local	1	–	–	≤ 3	3160 (b)	0 to 65+	0	6	6	1	10
Sans (1995) [67]	UK	Cohort	Moderate	1974–1984	Local	1	–	–	≤ 7.5	23,650	< 15	51	119	–	–	–
Wilkinson (1999) [75]	UK	Cohort	Low	1974–1991	National	11	–	–	≤ 2	7070 (b)	< 15	51 (b)	19	–	2	4
Yu (2006) [76]	Taiwan	Case-Control	High	1997–2003	Local	4	–	5	≤ 3	25	20–29	53	14	–	–	–
Zusman (2012) [57]	Israel	Cohort	Low	2000–2006	Local	1	1	1	≤ 0.5	14,050	30–64 (a)	48 (a)	–	–	–	9
All Studies	–	–	–	–	–	–	–	–	–	187,585	–	–	579	103	96	539

^a^LEVEL OF ADJUSTMENT | *Low* Typically adjusted for age and gender only, *Moderate* Adjusted for age, gender and at least one other demographic characteristic (i.e. income, ethnicity, or occupation); *High* Additionally adjusted for at least one lifestyle or genetic risk factor (i.e. BMI, smoking status, or family history)

^b^SOURCE OF EXPOSURE | *UP* Upstream (‘Refineries’), *MID* Midstream (‘Petrochemical Storage Facilities’), *DWN* Downstream (‘Petrochemical Plants’ and the ‘Organic Chemical Industry’)

^c^EXPOSED POPULATION | (a) = Author correspondence; (b) Estimated from the 1991 UK census, or the Global Rural-Urban Mapping Projects 30 arc-second grids (GRUMP v1, 2000)

^d^CASES | *LK* Leukaemia, *MM* Multiple Myeloma, *HL* Hodgkin Lymphoma, *NHL* Non-Hodgkin Lymphoma

Relative risks were obtained for 21 different population groups, seven of which are for males, four for females, and ten examined risk irrespective of gender. A majority reported risk across the general population, with only three of the population groups being entirely comprised of children (< 15 years of age). In terms of petrochemical activity, 9 of the population groups lived near to “upstream” refinery operations, 4 lived near to “downstream” facilities producing commercially marketable chemical products, and 8 lived alongside both forms of petrochemical activity. Fenceline communities were typically defined as living within 5 km of a petrochemical facility, although a few authors used an exposure threshold of up to 8 km in their analysis [55, 56, 67].

The quality of the existing literature was assessed via the Newcastle-Ottawa scale, which evaluates the risk of bias introduced by the selection of participants, their comparability to reference populations, and differing definitions of exposure (see Tables 4, 5). Most studies were of moderate to high quality (score ≥ 6), with only six cohort studies deemed to be of reduced quality. The following analysis controls for differences in quality, if heterogeneity is detected by the model diagnostics. Otherwise, variations in study design are considered to have minimal influence on the measured outcome. Where applicable, quality was included as a categorical moderator effect (1 = High, 0 = Low), with the revised risk ratio reflecting the model’s high-quality subgroup.

**Table 4:** Newcastle-Ottawa scale for assessing cohort study quality

Lead Author (Publication Year)	Selection (Max = 4)	Comparability (Max = 2)	Exposure (Max = 3)	Overall Quality (Max = 9)
Axelsson (2010) [6]	3	1	2	6
Barregard (2009) [68]	3	1	2	6
Beale (2010) [69]	2	2	2	6
Bulat (2011) [56]	3	1	1	5
Fazzo (2016) [48]	3	1	1	5
Lyons (1995) [54] ^a^	2	1	2	5
Pasetto (2012) [73] ^a^	3	2	2	7
Pekkanen (1995) [55]	2	1	1	4
Salerno (2013) [74]	3	1	1	5
Sans (1995) [67]	2	2	2	6
Wilkinson (1999) [75]	2	2	2	6
Zusman (2012) [57] ^a^	3	0	1	4

^a^Studies not included in the meta-analysis of leukaemia incidence

**Table 5:** | Newcastle-Ottawa scale for assessing case-control study quality

Lead Author (Publication Year)	Selection (Max = 4)	Comparability (Max = 2)	Exposure (Max = 3)	Overall Quality (Max = 9)
De Roos (2010) [70] ^a^	4	2	2	8
García-Pérez (2015) [71]	2	1	3	6
Linos (1991) [72]	3	2	2	7
Yu (2006) [76]	3	2	1	6

^a^ Studies not included in the meta-analysis of leukaemia incidence

### Leukaemia incidence

**Fig. 2:**
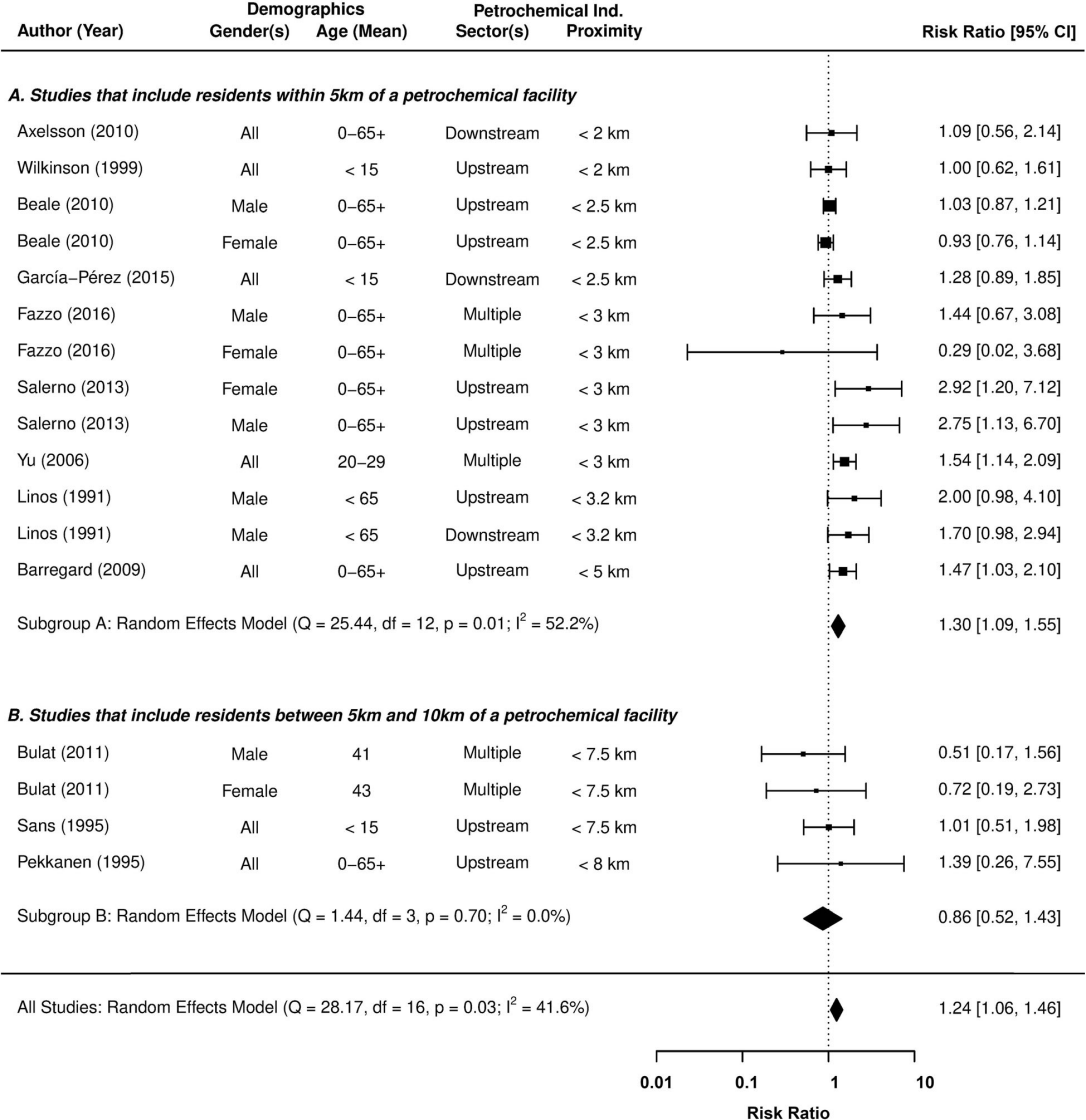
The association between residential exposure to petrochemical activity and the relative risk (RR) of Leukaemia incidence

Figure 2 provides pooled meta-analysis estimates on the relative risk (RR) of leukaemia incidence in fenceline communities, presented alongside the underlying observations reported by 12 different studies. None of the studies representing fenceline communities > 5 km from a petrochemical facility were observed to report an increased level of risk at the 95% confidence level. This observation was statistically confirmed by a subgroup meta-analysis of the three distant fenceline communities, whose pooled estimate found no evidence of increased risk (RR = 0.86; 95% CI = 0.52 to 1.43), or any between-study variation in effect size (I^2^ < 1%). Based on this evidence, the subsequent analysis focused on communities within 5 km of a petrochemical facility.

A pooled RR of 1.30 (95% CI = 1.09 to 1.55) indicates that leukaemia incidence is higher in fenceline communities no more than 5 km away from petrochemical activity. Beale et al’s [69] gender-based subpopulations within the US state of Utah collectively provide the largest, but by no means overriding influence on the pooled estimate (31.5% of the weight). Meanwhile, the lowest level of influence (0.5% of the weight) is attributed to the experience of female residents from Sicily [48]. These meta-analysis weightings are reflective of uncertainty in the measurements of RR reported by the underlying studies. Still, moderate levels of heterogeneity were detected between the study group risk estimates (I^2^ = 52.2%), which must be accounted for, in order to test the robustness of these findings.

A series of meta-regressions were conducted to identify and control for potential causes of heterogeneity in the risk of leukaemia incidence for fenceline communities (see Table 6). The influence of categoric and continuous moderator effects were investigated by a Wald-type chi-square test (Q_M_), where *p* > 0.05 rejects the null hypothesis of no relationship between the effect size and moderator variable(s). Any remaining sources of heterogeneity found in the model’s residuals are considered of negligible influence, if the I^2^ test returns a value below 25%, and or Cochran’s Q rejects its null hypothesis (Q_E_*p*-value > 0.05).

**Table 6:** Pooled estimates on the relative risk (RR) of leukaemia incidence within 5 km of a petrochemical facility, moderated by different characteristics

Assessment	Characteristics	Heterogeneity Tests			Pooled Relative Risks (95% CI)
		I^2^ (%)	Q_E_ (p-value) ^c^	Q_M_ (p-value) ^c^	
Base Model	–	52.18	0.01	–	–
Demographic	1.1. Gender • All (*n* = 5) • Male (*n* = 5) • Female (*n* = 3)	47.98	0.03	0.67	–
	1.2. Participants • Children (*n* = 2) • General Population (*n* = 11)	57.63	0.01	0.51	–
Diagnosis	2.1. Classification Scheme • ICD 7–10 (*n* = 8) • ICD for Oncology (*n* = 5)	49.48	0.02	0.37	–
	2.2. Classification Scheme • ICD 7–8 (*n* = 3) • ICD 9–10 (*n* = 5) • ICD for Oncology (*n* = 5)	56.71	0.02	0.73	–
Exposure	3.1. Petrochemical Sector • Upstream (*n* = 7) • Downstream (*n* = 3) • Combination (*n* = 3)	52.99	0.02	0.85	–
	3.2. Maximum Distance • 3 km (*n* = 10) • 3.1 to 5 km (*n* = 3)	41.98	0.05	0.12	–
Geography	4. Continent • Europe (*n* = 8) • North America (*n* = 4) • Asia (*n* = 1)	31.67	0.09	0.24	–
Quality ^d^	5.1. Newcastle-Ottawa Score • Low (*n* = 4) • High (*n* = 9)	45.10	0.04	0.07	–
	5.1.1. Participant Selection • Low (*n* = 4) • High (*n* = 9)	< 0.01	0.60	< 0.01	Low: 1.01 [0.90 to 1.14] High: 1.58 [1.32 to 1.90]
	5.1.2. Study Comparability • Low (*n* = 7) • High (*n* = 6)	45.07	0.04	0.20	–
	5.1.3. Outcome Assessment • Low (*n* = 5) • High (*n* = 8)	33.38	0.12	0.03	Low: 1.71 [1.25 to 2.35] High: 1.15 [0.98 to 1.37]
Temporal	6.1. Study Start • 1965 to 1974 (*n* = 3) • 1975 to 1984 (*n* = 3) • 1985 to 1994 (*n* = 2) • 1995 to 2004 (*n* = 5)	< 0.01	0.42	< 0.01	1965/74: 1.02 [0.91 to 1.15] 1975/84: 1.21 [1.09 to 1.34] 1985/94: 1.43 [1.23 to 1.65] 1995/04: 1.69 [1.36 to 2.11] 2005/14: 2.00 [1.48 to 2.70] ^b^
	6.2. Study Start (+ 1 Year) ^a^	< 0.01	0.28	< 0.01	1971: 1.00 [0.88 to 1.14] 1981: 1.20 [1.09 to 1.33] 1991: 1.44 [1.23 to 1.69] 2001: 1.73 [1.36 to 2.22] 2011: 2.08 [1.48 to 2.93] ^b^
Mixed Effect	5.1.1. Participant Selection, and 6.2 Study Start (+ 1 Year) ^a^	< 0.01	0.63	< 0.01	1971: 1.38 [1.02 to 1.87] 1981: 1.49 [1.21 to 1.84] 1991: 1.61 [1.34 to 1.93] 2001: 1.74 [1.36 to 2.22] 2011: 1.88 [1.32 to 2.67] ^b^

^a^ Continuous variable

^b^ Predicted risk

^c^ Wald type Chi-Squared tests: *Q*_*E*_ Test for Residual Heterogeneity, *Q*_*M*_ Test of Moderators

^d^ Newcastle-Ottawa Score: Total (L = 0–5, H = 6–9), Selection (L = 0–2, H = 3–4), Comparability (L = 0–1, H = 2), Assessment (L = 0–1, H = 2–3)

In terms of demographic attributes, no apparent differences were found in relation to gender or age, although few studies specifically examine leukaemia incidence in children [71, 75]. Considering that heavy industries are traditionally male-dominated forms of employment, it could have been argued that increased levels of risk are associated only with occupation and not residential exposure, but this is not the case.

The meta-regressions identified no differences in risk from residential exposure to a specific sector of the petrochemical industry, be that from an upstream refinery or downstream manufacturer of petroleum-based goods (Q_M_*p*-value = 0.85). The petrochemical industry also appears to pose a common risk, regardless of any differences in operational practices at either a continental (Q_M_ p-value = 0.24) or national (Q_M_ p-value = 0.45) level of analysis. The presence of confounding caused by revisions to the classification schemes used to diagnose blood cancers was then tested for, the impact of which appears to be negligible (Q_M_*p*-value = 0.37 and 0.73).

Heterogeneity between the individual study effects appears to be linked to aspects of study quality, measured by the three underlying themes of the Newcastle-Ottawa scale. Virtually all traces of heterogeneity were removed when controlling for differences in quality, based on the “Participant Selection” process of each study (I^2^ < 1%). The participant selection processes were critiqued based on the format of underlying data (i.e. record linkage or self-reports), its validity, and the representativeness of sampling procedures.

The pooled RR of leukaemia incidence in fenceline communities increased to 1.58 (95% CI = 1.32 to 1.90), after controlling for participant selection. The meta-regression outputs confirm that any variation in study design is sufficiently accounted for (I^2^ < 1%, Q_E_*p*-value = 0.60), and no publication bias exists amongst the identified studies (Egger’s *p*-value = 0.67).

Another consideration is the likely change in quantity and perhaps even the composition of pollutants emitted from petrochemical facilities over time, with manufacturing processes and abatement technology developing in response to evolutions in the environmental legislation. The impact of a changing petrochemical landscape on reported risk, was therefore evaluated by controlling for the initial reporting year of each study. These temporal meta-regression models also comprehensively explained the variation in risk between studies (I^2^ < 1%), and surprisingly reported an increase in risk with time (see Table 6).

**Fig. 3:**
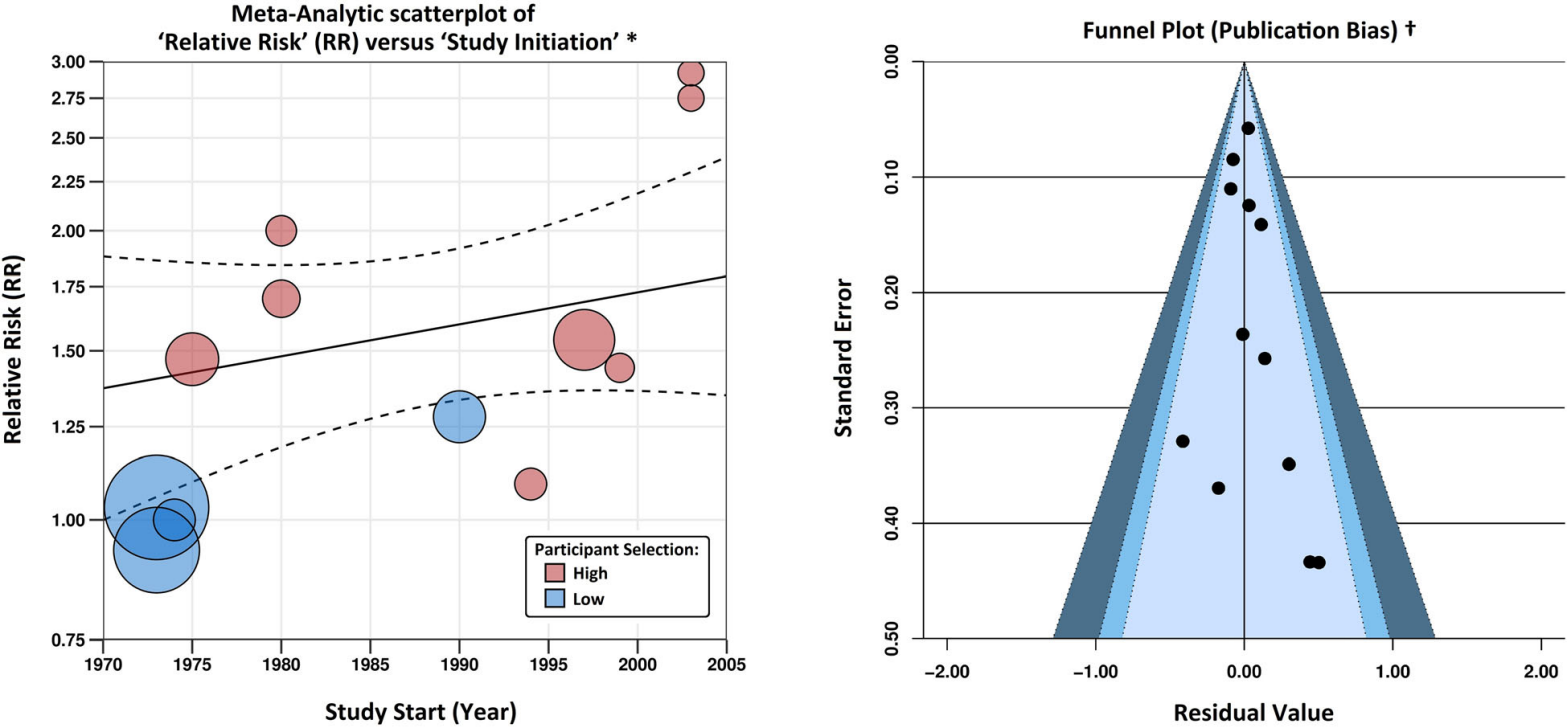
Meta-regression outputs, illustrating the relationship between Relative Risk (RR) of leukaemia incidence and the initial reporting year of each study, while controlling for study quality. The contour-enhance funnel plot is used to check for the impact of unpublished literature, and the significance of individual studies

A final meta-regression was created, which controlled for quality (in terms of participant selection) and the initial reporting year of each study (see Table 6 and Fig. 3). The model shows no signs of heterogeneity (I^2^ < 1%) or publication bias (Egger’s p-value = 0.93), and the highest rates of risk are still found in the most recent population studies. The RR of leukaemia incidence from petrochemical operations in 2011 is estimated at 1.88 (95% CI = 1.32 to 2.67), compared to 1.38 (95% CI = 1.02 to 1.87) in 1971. Although it is unlikely that an increasingly regulated industry will pose the greater risk, we can conclude that the potential risk of petrochemical operations certainly has not diminished over time..

### Other incidences of Haematological malignancy

The systematic review then evaluated the epidemiological evidence base of the three remaining categories of haematological malignancy, in the form of Non-Hodgkin’s Lymphoma (NHL), Hodgkin’s Lymphoma (HL), and Multiple Myeloma (MM).

**Fig. 4:**
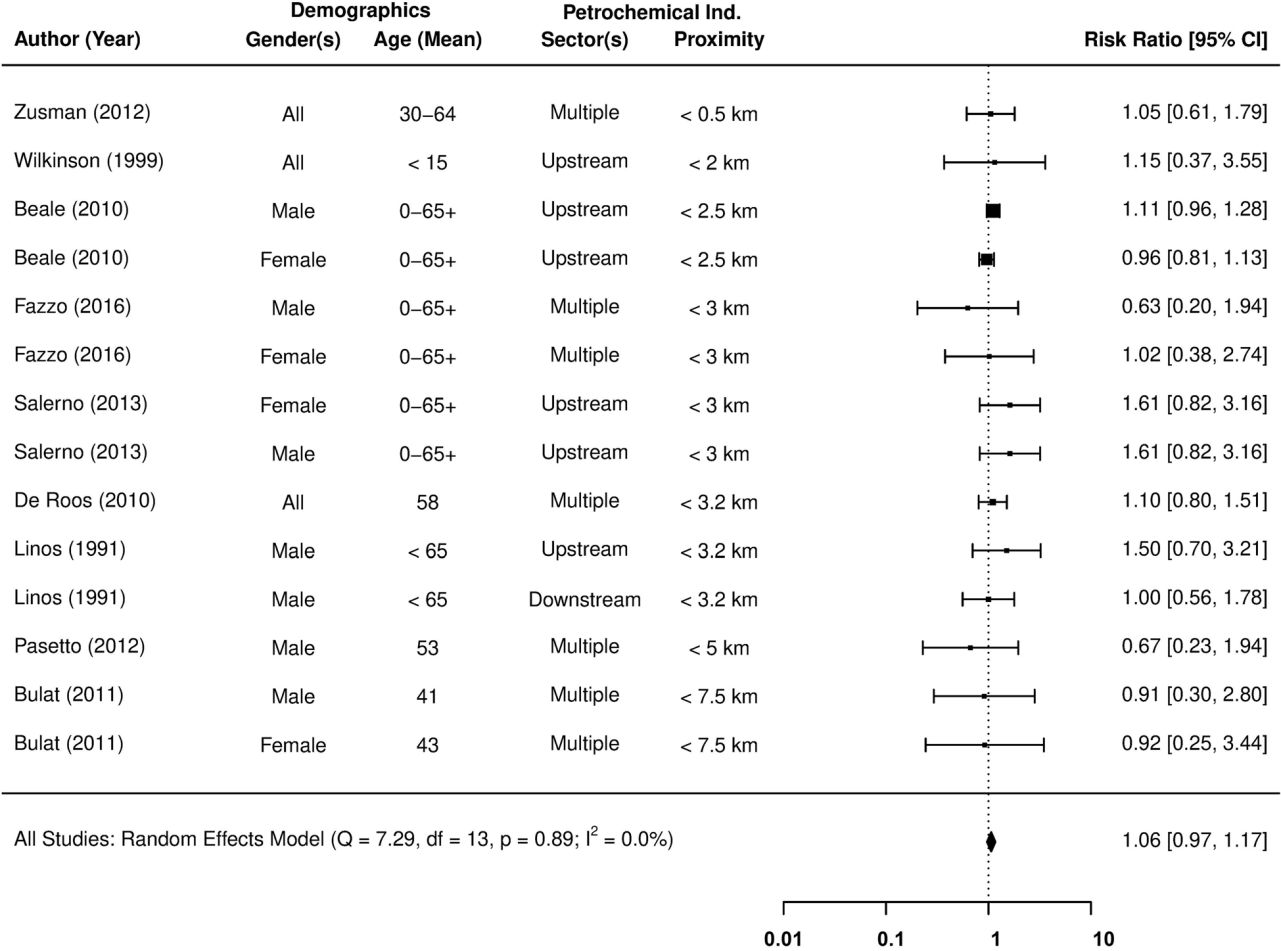
The association between residential exposure to petrochemical activity and the relative risk (RR) of Non-Hodgkin’s Lymphoma incidence

Figure 4 presents the underlying observations of 9 different studies and the pooled meta-analysis RR estimate of Non-Hodgkin’s Lymphoma incidence in fenceline communities. The model outputs confirm that the study effects are homogeneous, even when including studies that define the upper limit of fenceline communities at 7.5 km (I^2^ < 1%, Q_E_*p*-value = 0.89). The creation of a symmetrically distributed funnel plot (see Additional file 1) and its two null hypothesis tests also indicate an absence of publication bias in the reported effect sizes (Egger’s p-value = 0.85). The existing epidemiological evidence indicates that people living within 7.5 km of a petrochemical facility are at greater risk of developing Non-Hodgkin’s Lymphoma (RR = 1.06; 95% CI = 0.97 to 1.17). However, further research is required to verify these findings.

**Fig. 5:**
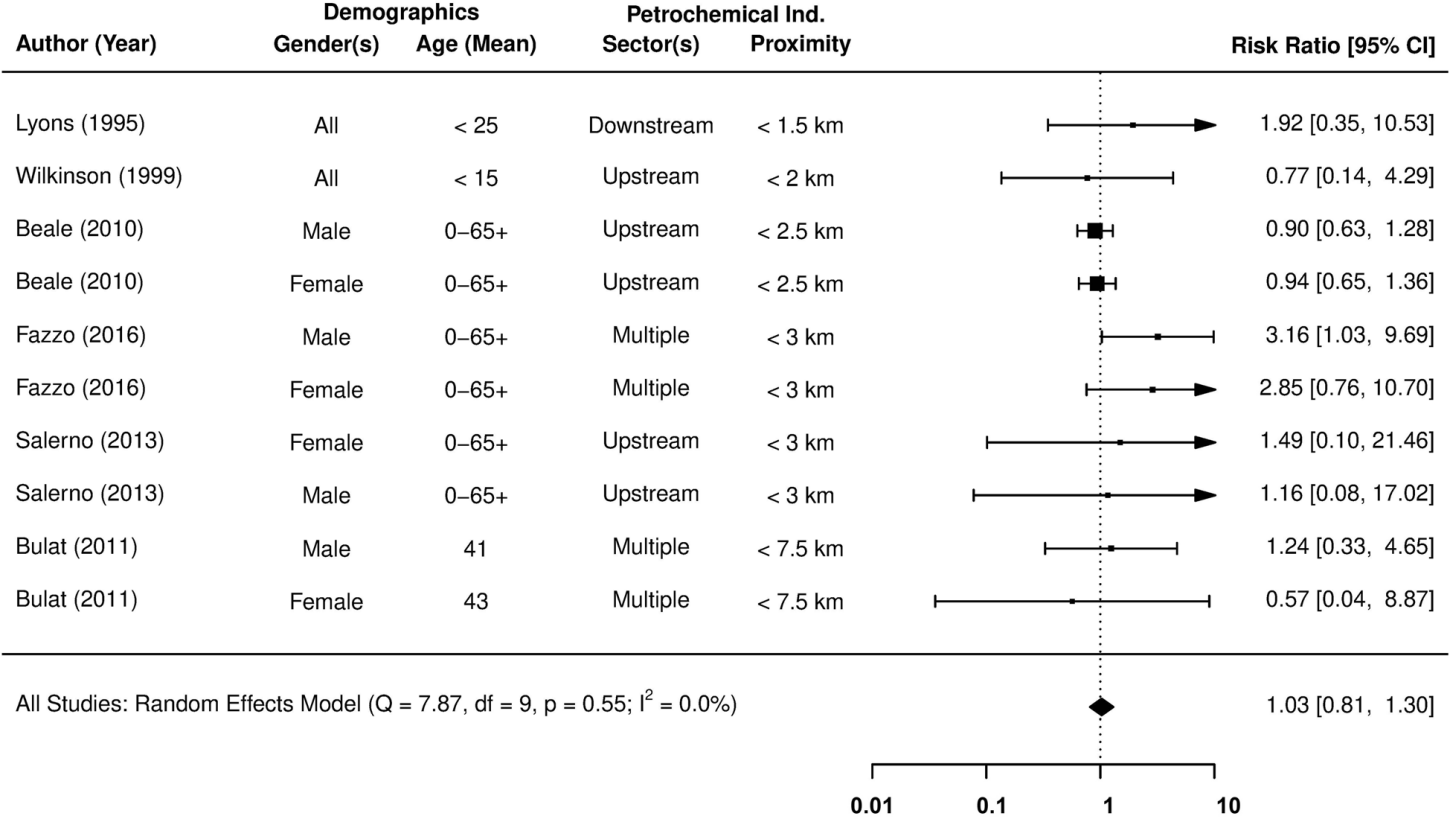
The association between residential exposure to petrochemical activity and the relative risk (RR) of Hodgkin’s Lymphoma incidence

Figure 5 presents the underlying observations of 9 different studies and the pooled meta-analysis RR estimate of Hodgkin’s Lymphoma incidence in fenceline communities. Although the study effects appear to be homogeneous (I^2^ < 1%, Q_E_ p-value = 0.55), a somewhat asymmetric funnel plot and close rejection Egger’s test (p-value = 0.16) may indicate publication bias.

**Fig. 6:**
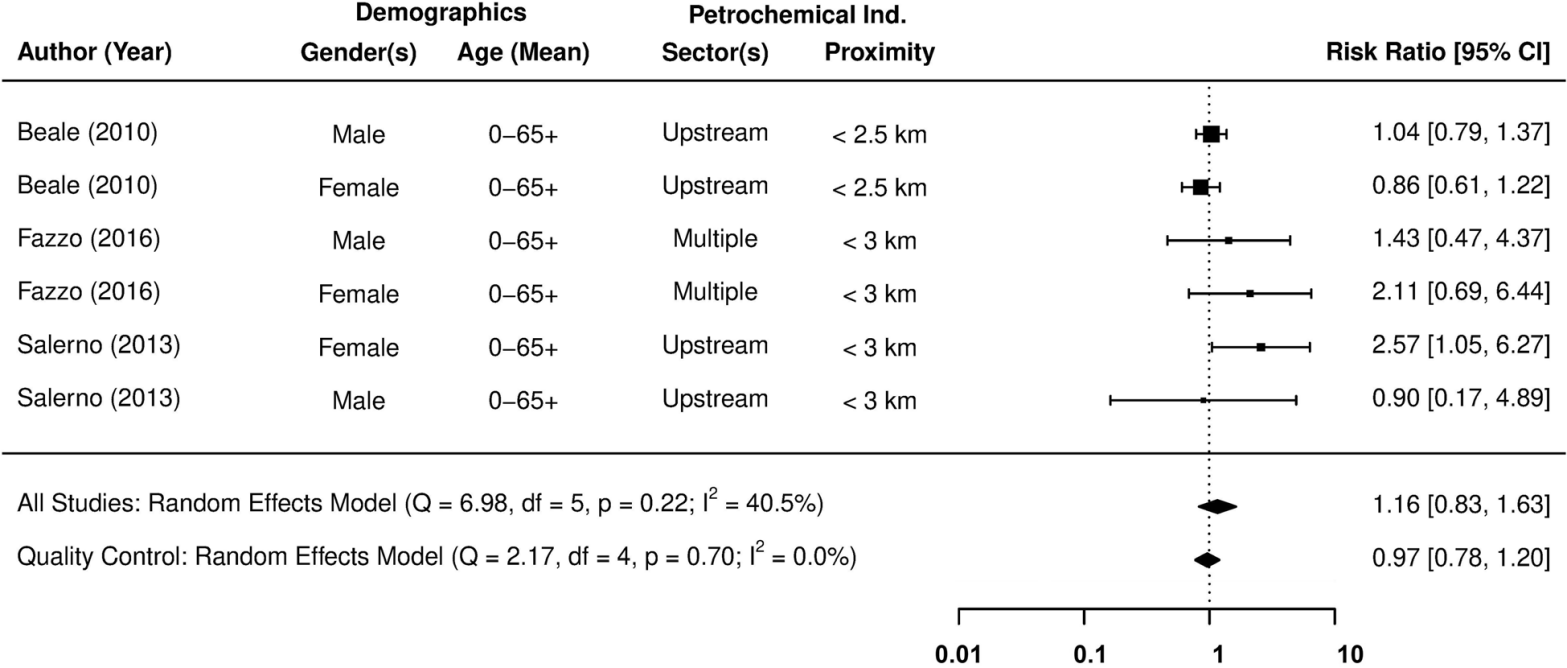
The association between residential exposure to petrochemical activity and the relative risk (RR) of Multiple Myeloma incidence

Finally, a pooled RR estimate of Multiple Myeloma incidence in fenceline communities was constructed from gender-specific effects, reported by 3 different studies (see Fig. 6). Whilst the meta-analysis of the raw effect sizes returned a positive estimate with high levels of uncertainty (RR = 1.16, 95% CI = 0.83 to 1.63), there were issues of heterogeneity between the studies (I^2^ ≈ 41%). The variation in effect size appeared to be linked to study quality, as measured by the Newcastle-Ottawa scale overall score (I^2^ < 1%). The resulting revised model found no clear link between Multiple Myeloma and residential exposure to the petrochemical industry (RR = 0.97, 95% CI = 0.78 to 1.20).

### Case-study: Louisiana’s petrochemical corridor

Louisiana is located within the southeastern United States, where the Mississippi River meets with the Gulf of Mexico. It is considered one of the most toxic states, annually discharging 7.2 t of hazardous waste per capita, and accounting for 12.5% of the country’s hazardous waste from only 6.5% of the nation’s chemical facilities [77, 78]. The ‘National Cancer Institute’ currently recognises that age-adjusted rates of overall cancer incidence in Louisiana, are 7.3% above the national estimate of 448 cases per 100,000 persons [79]. Along the Louisiana stretch of the Mississippi, there are several densely packed industrial zones which form a petrochemical corridor from Baton Rouge to New Orleans, often referred to as “Cancer Alley”.

Table 7 presents the number of newly diagnosed leukaemia cases in each parish, that are attributed to residential exposure from the petrochemical industry along the Louisiana stretch of the Mississippi River. Approximately 188,075 residents from 12 parishes live within 5 km of a ‘highly’ polluting petrochemical facility. Over 40% of the residents from the sparsely populated parishes (< 50,000 persons) of Iberville and St. Bernard live in fenceline communities. By count, the parish of East Baton Rouge has the largest number of residents in fenceline communities (*n* = 82,554).

**Table 7:** Estimated population attributable factor and Leukaemia Incidence cases for Louisiana residents living within 5 km of a petrochemical facility along the Mississippi River (2011–15)

Parish Name	Population			Age-Adjusted Leukaemia Incidence (2011–15)		Population Attributable Fraction (%)**	Attributable Leukaemia Cases (2011–15)
	Exposed (N)	Total (N)	Exposed (%)	Annual Cases Per 100,000 *	5-Year Count (N)		
(1) East Baton Rouge	82,554	440,046	19%	13.2 [11.7–14.8]	290.4 [257.4–325.6]	10% [6–14%]	28.5 [14.6–47.0]
(2) Orleans	37,360	343,573	11%	11.2 [9.7–12.9]	192.4 [166.6–221.6]	6% [3–9%]	11.4 [5.6–19.8]
(3) St. Bernard	22,542	35,887	63%	8.4 [4.8–13.5]	15.1 [8.6–24.2]	27% [17–36%]	4.0 [1.4–8.8]
(4) St. Charles	13,730	52,745	26%	11.8 [7.9–16.9]	31.1 [20.8–44.6]	13% [8–19%]	4.1 [1.6–8.5]
(5) Iberville	13,223	33,387	40%	14.0 [9.0–20.9]	23.4 [15.0–34.9]	19% [11–26%]	4.4 [1.7–9.2]
(6) St. John the Baptist	9796	45,914	21%	12.0 [7.7–17.8]	27.6 [17.7–40.9]	11% [6–16%]	3.0 [1.1–6.6]
(7) West Baton Rouge	4553	23,774	19%	13.9 [13.4–14.4] †	16.5 [15.9–17.1]	10% [6–15%]	1.7 [0.9–2.5]
(8) Ascension	1833	107,208	2%	12.3 [9.2–15.9]	65.9 [49.3–85.2]	1% [1–2%]	0.7 [0.3–1.3]
(9) St. James	1519	22,100	7%	13.9 [13.4–14.4] †	15.4 [14.8–15.9]	4% [2–6%]	0.6 [0.3–0.9]
(10) Plaquemines	940	23,031	4%	13.9 [13.4–14.4] †	16.0 [15.4–16.6]	2% [1–4%]	0.4 [0.2–0.6]
(11) Lafourche	25	96,301	< 0.1%	15.3 [12.1–19.1]	73.7 [58.3–92.0]	0% [0–0%]	0.0 [0.0–0.0]
(12) Jefferson	0	432,415	0%	13.6 [12.2–15.2]	294.0 [263.8–328.6]	0% [0–0%]	0.0 [0.0–0.0]

*Age-Adjusted Leukaemia Incidence rates obtained from by the National Cancer Institute (https://statecancerprofiles.cancer.gov/data-topics/incidence.html)

** Leukaemia Incidence relative risk (RR) = 1.58 [1.32–1.9]

†Use of the Louisiana State cancer rate, in parishes where incidence rates have been censored and conditions with a low occurrence (< 3 cases per annum)

**Fig. 7:**
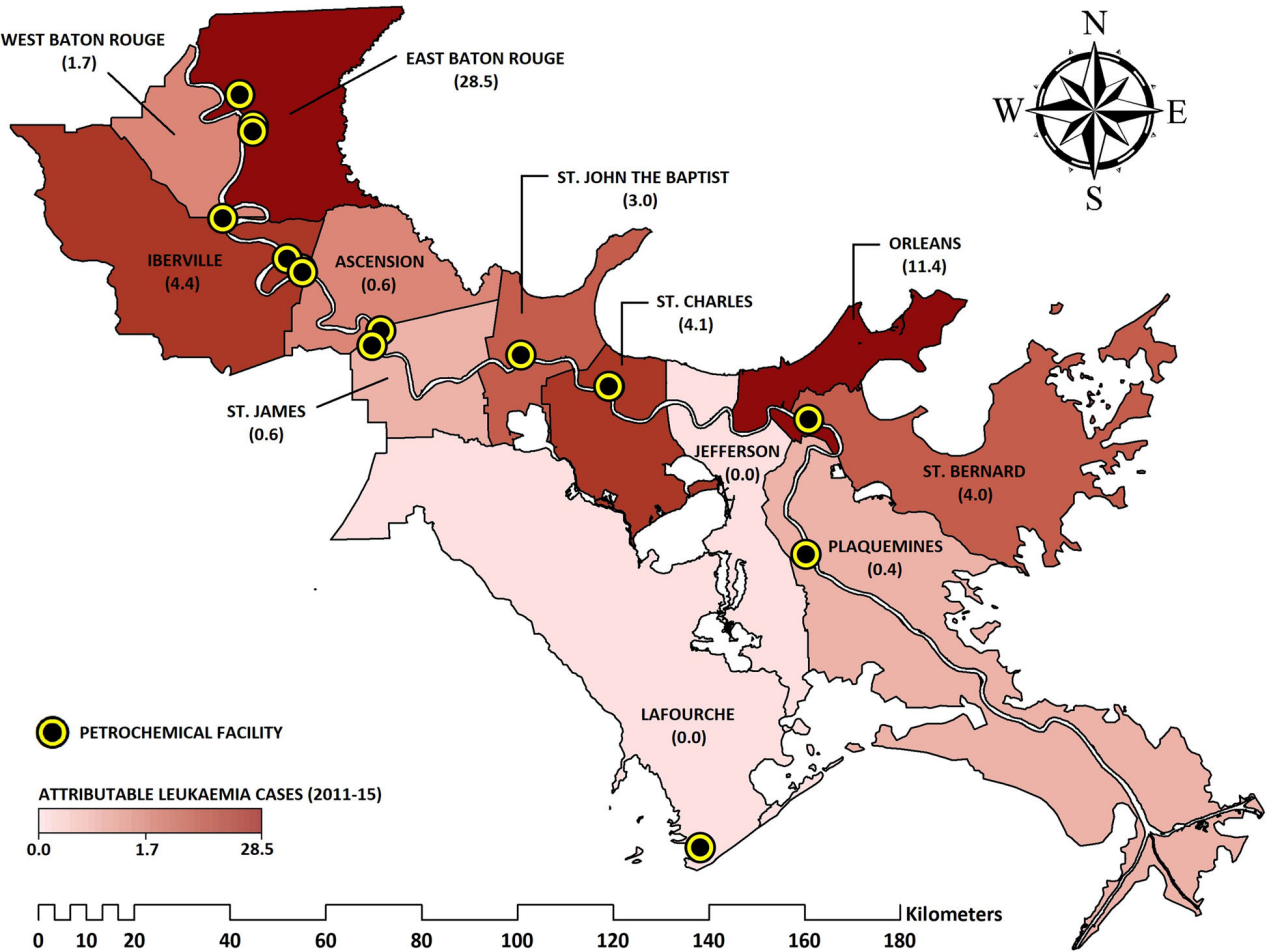
Estimated cases of Leukaemia Incidence in Louisiana riverside parishes, which are attributable to residential exposure to the petrochemical industry (2011–15)

It is estimated that the petrochemical industry is accountable for 58.7 (27.8 to 105.1) new cases of leukaemia in fenceline communities along this river corridor, over the 5-year period of 2011–15, when applying our meta-analysis risk ratio to parish level health data. Figure 7 displays the number of attributable cancer incidence cases for each parish, with respect to these ‘highly’ polluting petrochemical facilities.

## Discussion

### Principal findings

The systematic review and meta-analysis identified 16 unique studies which collectively record the incidence of haematological malignancies across 187,585 residents living in close proximity to petrochemical sites. Across varied geographical contexts and covering a period of analysis between 1960 and 2011, the review found that those living within 5 km of a petrochemical facility have a 30% higher risk of developing leukaemia than residents from communities with no petrochemical activity. The enhanced risk for fenceline communities applied to upstream, midstream and downstream petrochemical operations.

The analysis indicated that heterogeneity between the 16 studies may be explained by differences in study quality, notably in relation to the participant selection process of the research. The meta-analysis also found temporal differences, with the most recent exposure studies reporting the highest rates of leukaemia risk in fenceline communities. Given the increasingly regulated nature of the petrochemical industry, this was an unexpected and surprising finding. It is plausible that the earliest studies investigated sites representative of the wider industry, with later research guided by a wealth of anecdotal evidence and primarily focusing on the most polluting operations.. Thus, we do not suggest that the risk of developing leukaemia in fenceline communities is becoming greater, but rather that the risk for residents living close to petrochemical sites persists.

The incidences of Non-Hodgkin’s Lymphoma (NHL), Hodgkin’s Lymphoma (HL), and Multiple Myeloma (MM) in fenceline communities remain uncertain and further research is required to determine the risk of developing these blood cancers in fenceline communities of petrochemical sites.

Building on the meta-analysis, we estimate the potential impact of the petrochemical industry on leukaemia incidence in Louisiana’s Mississippi Valley, between 2011 and 2015. Nineteen petrochemical facilities in the state of Louisiana, were currently found to release emissions of a BTEX compound above or equal to expected industry levels in 1987, fifteen of which are located close to the Mississippi. It is estimated that 188,075 residents from 12 parishes along Louisiana’s Mississippi Valley, known as ‘Cancer Alley’, live within 5 km of a ‘highly’ polluting petrochemical facility. The findings suggest that the petrochemical industry is accountable for 58.7 new cases of leukaemia in fenceline communities in ‘Cancer Alley’, between 2011 and 2015. This estimate is thought to be conservative, as we only considered the impact from 15 out of 54 identifiable petrochemical facilities from these 12 parishes (28%), based on the criteria of ‘high’ levels of BTEX emissions under normal operating conditions.

### Comparison with other studies

The findings from this meta-analysis support existing occupational research on the incidence of haematological malignancies among workers in the petrochemical industry, which observed close associations between exposure to toxic pollutants and the development of blood cancers across the upstream, midstream and downstream sectors of the industry [24, 30, 34, 35]. The higher occupational risks also applied to workers exposed to low levels of BTEX concentrations [10–12]. Along the same lines, the analysis in this paper indicates that also residents of fenceline communities in close proximity to petrochemical sites carry a greater risk of developing leukaemia.

The findings from this meta-analysis extend previous epidemiological research on associations between residential exposure to releases from petrochemical facilities and health risk. It adds to Lin et al’s [7, 8] meta-analyses of lung cancer incidence and mortality in residential populations sin close proximity to petrochemical industrial clusters. Despite cleaning up and the development of emissions abatement technologies in recent years, petrochemical industrial sites remain closely associated with substantial toxic and hazardous releases and continue to pose a risk to the health of fenceline communities.

### Study strengths and limitations

This is the first known meta-analysis of blood cancer risk in fenceline communities next to petrochemical industrial sites. The meta-analysis investigated the association between incidences of haematological malignancies and residential exposure to releases from the petrochemical industry. The analysis of incidence rates rather than mortality rates can reduce the bias caused by other factors which may affect blood cancer survival rates (e.g. healthcare quality) [7, 80].

The meta-analysis adopted a rigorous study selection process, including a comprehensive search of relevant academic databases and a strict application of criteria in selecting studies. All incident cases in the identified studies were diagnosed by a medical professional and coded in accordance to the International Classification of Diseases (ICD). Furthermore, studies were only included if they reported health outcomes under normal operating conditions (not emergency events), and if they provided a clear definition of fenceline communities, thus avoiding the limitations of Lin et al’s [7, 8] meta-analytical reports on the petrochemical industry. In terms of analysis, the extraction of a single effect size from each study minimised the risk of dependency between effect sizes [46, 47], while allowing for the investigation of moderating influences. Indeed, a number of potential causes of heterogeneity in the risk of leukaemia incidence at fenceline communities were controlled for and investigated in the analysis (e.g. study quality, age, gender).

However, given that the identified 16 studies are clustered in three geographical regions, there may exist concerns over generalisability in this meta-analysis. Exposure levels and health conditions may be affected by geographical differences in environmental law, housing regulations, and access to healthcare, yet we observed no unexplained discrepancies between the European and North American literature. Moreover, there are difficulties in assessing the health impacts of pollution from the petrochemical industry in one discrete time period, as these studies did. Blood cancers may develop years after exposure and the slow temporal dimensions of pollution may be a possible explanation for why the later studies indicated higher risk levels of leukaemia in fenceline communities. Additionally, the quality assessments carried out using the Newcastle-Ottawa scale are subjective and limited, in that we can never know the full extent of the studies’ quality and can only judge by the information provided by the investigators.

Furthermore, other risk factors, notably socioeconomic status, were often not universally adjusted for by the selected studies, which may influence the findings [81]. Although, by controlling for differences in participant selection (i.e. representativeness) we were able to account for between study heterogeneity. Additionally, it is difficult to disentangle residential risk from occupational risk. Some of the residents living within 5 km of the petrochemical site are likely to be employees and, accordingly, it is methodologically challenging to limit exposure to solely residential. Still, occupational exposure research has reported the risk of lung cancer mortality and hospital discharges for progressive lung diseases to be 71 and 40% higher in petrochemical employees from fenceline communities, compared to employees that commuted, respectively. Based on the findings of our meta-analysis, the risk from residential exposure is also likely to exist in relation to leukaemia [73]. However, there remains a need for further research which accounts for, and reduces the influence of, occupational factors in assessing residential exposure. Equally, it is problematic to specifically associate higher incidences of leukaemia with a particular petrochemical site, given that these are often located in industrial complexes in which other manufacturing processes are occurring.

All studies examined in this systematic review indirectly measured exposure to a petrochemical facility in terms of proximity, however, exposure can only be truly confirmed by direct measurements of air quality and biospecimens (i.e. a complete exposure pathway). Only two of our identified studies provided exposure measurements, which crudely summarised the areas of high and low exposure [6, 68], and this is clearly an area in need of further development. Still, it remains extremely challenging to precisely measure and apportion concentrations to a specific source, let alone accurately measure personal levels of exposure. Given these limitations, proximity remains a suitable and low-cost indication of exposure outcomes, which should then be confirmed on an individual basis.

## Conclusions

The meta-analysis provides evidence for a higher risk of leukaemia development in individuals living near a petrochemical facility, due to exposure to toxic and hazardous emissions. Meta-analysis findings such as these can act as evidence base for public health policy priorities, including the setting up of preventive strategies and standards, and the tightening of regulations on toxic and hazardous pollutants from the petrochemical industry. Our findings can also be used to assist legal cases of grassroots environmental movements whose research capacities may be limited or hindered by the power and influence of industry.

## Supplementary information

**Additional file 1.**

## Data Availability

The supporting data and model scripts are available online, through the BMC Environmental Health repository.

## Abbreviations

BTEX: Volatile organic compounds, which may include benzene, toluene, ethylbenzene, and or xylene
HL: Hodgkin Lymphoma
IARC: International Agency for Research on Cancer
LK: Leukaemia
MM: Multiple Myeloma
MOOSE: Meta-analyses Of Observational Studies in Epidemiology
NHL: Non-Hodgkin Lymphoma
OR: Odds Ratio
PAF: Population Attributable Fraction
PRISMA: Preferred Reporting Items for Systematic reviews and Meta-Analyses
RR: Relative Risk
SE: Standard Error
SIR: Standardised Incidence Ratio
TRI: Toxic Release Inventory
WHO: World Health Organisation
