# Supplementary material for: A systematic review and meta-analysis of haematological malignancies in residents living near petrochemical facilities
Source: Environ Health. 2020 May 19;19:53. doi: 10.1186/s12940-020-00582-1 (PMC7236944; doi:10.1186/s12940-020-00582-1)
Supplement: Supplementary file 1 — Additional file 1. [file 12940_2020_582_MOESM1_ESM.docx]

**- ONLINE APPENDICES -**

**APPENDIX 1:** MOOSE Checklist for Meta-analyses of Observational Studies

**APPENDIX 2:** PRISMA Checklist for the transparent reporting of systematic reviews and meta-analyses

**APPENDIX 3:** Details of the database search strategy, conducted on 14/11/18

**APPENDIX 4:** Newcastle-Ottawa Quality Assessment Scale (Cohort Studies)

**APPENDIX 5:** Newcastle-Ottawa Quality Assessment Scale (Case-Control Studies)

**APPENDIX 6:** High resolution dasymetric modelled population counts of Louisiana, aggregated into 5x5km grids (Source: Dmowska & Stepinski 2014)

**APPENDIX 7:** Funnel plots for the association between residential exposure to petrochemical activity and the relative risk (RR) of Non-Hodgkin’s Lymphoma, Hodgkin’s Lymphoma and Multiple Myeloma incidence

**APPENDIX 1 | MOOSE checklist for the Meta-analyses of Observational Studies**

| **Item** | **Recommendation** | **Report Section** |
| --- | --- | --- |
| **Reporting of background should include:** | | |
| 1 | Problem definition | 1 |
| 2 | Hypothesis statement | Objective |
| 3 | Description of study outcome(s) | Table 2 |
| 4 | Type of exposure or intervention used | 2.2 |
| 5 | Type of study designs used | 2.2 |
| 6 | Study population | 2.2 |
| **Reporting of search strategy should include:** | | |
| 7 | Qualifications of investigators | Authors |
| 8 | Search strategy, including time period included in the synthesis and key words | 2.1 & Appendix 3 |
| 9 | Effort to include all available studies, including contact with authors | 2.3 |
| 10 | Databases and registries searched | 2.1 |
| 11 | Search software used, name and version, including special features used | 2.1 |
| 12 | Use of hand searching (e.g. reference lists of obtained articles) | 2.1 |
| 13 | List of citations located and those excluded, including justification | Figure 1 & 2.2 |
| 14 | Method of addressing articles published in languages other than English | 2.1 |
| 15 | Method of handling abstracts and unpublished studies | 3 |
| 16 | Description of any contact with authors | 2.3 |
| **Reporting of methods should include:** | | |
| 17 | Description of relevance or appropriateness of studies assembled for assessing the hypothesis | 2.2 |
| 18 | Rationale for the selection and coding of data (e.g. sound clinical principles or convenience) | 2.3 |
| 19 | Documentation of how data were classified and coded (e.g. blinding and interrater reliability) | 2.3 |
| 20 | Assessment of confounding (e.g. comparability of cases and controls) | 2.4 |
| 21 | Assessment of study quality (e.g. blinding of quality assessors, stratification or regression on predictors) | 2.3 |
| 22 | Assessment of heterogeneity | 2.5 |
| 23 | Description of statistical methods (e.g. use of fixed or random effects models, whether the chosen models account for predictors of study results, or cumulative meta-analysis) in enough detail for replication | 2.5 |
| 24 | Provision of appropriate tables and graphics | Figure 1 |
| **Reporting of results should include:** | | |
| 25 | Graphic summarising individual study estimates and overall estimate | Figures 2, 4-6 |
| 26 | Table giving descriptive information for each study included | Table 2 |
| 27 | Results of sensitivity testing (e.g. subgroup analysis) | Table 3 |
| 28 | Indication of statistical uncertainty of findings | Figures 2, 4-6 |
| **Reporting of discussion should include:** | | |
| 29 | Quantitative assessment of bias (e.g. publication bias) | 3.2 |
| 30 | Justification for exclusion (e.g. exclusion of non-English language citations) | 4.3 |
| 31 | Assessment of quality of included studies | 3.2 |
| **Reporting of conclusions should include:** | | |
| 32 | Consideration of alternative explanations for observed results | 4.3 |
| 33 | Generalisation of the conclusions (appropriate for the data presented and the domain of the literature) | 4.1 |
| 34 | Guidelines for future research | 4.3 |
| 35 | Disclosure of funding source | Acknowledgements |

**Adapted From:** Stroup D, Berlin J, Morton S, Olkin I, Williamson G, Rennie D, Moher D, Becker B, Sipe T & Thacker S (2000). Meta-analysis Of Observational Studies in Epidemiology (MOOSE): A Proposal for Reporting. *JAMA*, 283(15), pp.2008-2012. <http://statswrite.eu/pdf/MOOSE%20Statement.pdf>

**APPENDIX 2 | PRISMA Checklist for the transparent reporting of systematic reviews and meta-analyses**

| **Item** | **Recommendation** | **Report Section** |
| --- | --- | --- |
| **TITLE:** | | |
| 1 | Identify the report as a systematic review, meta-analysis, or both. | Title |
| **ABSTRACT** | | |
| 2 | Provide a structured summary including, as applicable: background; objectives; data sources; study eligibility criteria, participants, and interventions; study appraisal and synthesis methods; results; limitations; conclusions and implications of key findings; systematic review registration number | Abstract |
| **INTRODUCTION** | | |
| 3 | Describe the rationale for the review in the context of what is already known. | 1 |
| 4 | Provide an explicit statement of questions being addressed with reference to participants, interventions, comparisons, outcomes, and study design (PICOS). | 1 |
| **METHODS** | | |
| 5 | Indicate if a review protocol exists, if and where it can be accessed (e.g. Web address), and, if available, provide registration information including registration number. | 2.1 |
| 6 | Specify study characteristics (e.g. PICOS, length of follow-up) and report characteristics (e.g. years considered, language, publication status) used as criteria for eligibility, giving rationale. | 2.2 & appendix 3 |
| 7 | Describe all information sources (e.g., databases with dates of coverage, contact with study authors to identify additional studies) in the search and date last searched. | 2.1 & Appendix 3 |
| 8 | Present full electronic search strategy for at least one database (including limits), so that it could be repeated. | Appendix 3 |
| 9 | State the process for selecting studies (e.g. screening, eligibility, included in systematic review, and, if applicable, included in the meta-analysis). | 2.2 & Figure 1 |
| 10 | Describe method of data extraction from reports (e.g. piloted forms, independently, in duplicate) and any processes for obtaining and confirming data from investigators. | 2.3 |
| 11 | List and define all variables for which data were sought (e.g. PICOS, funding sources) and any assumptions and simplifications made. | 2.3 |
| 12 | Describe methods used for assessing risk of bias of individual studies (including specification of whether this was done at the study or outcome level), and how this information is to be used in any data synthesis. | 2.3 |
| 13 | State the principal summary measures (e.g. risk ratio, difference in means). | 2.4 |
| 14 | Describe the methods of handling data and combining results of studies, if done, including measures of consistency (**see I2**) for each meta-analysis. | 2.4 |
| 15 | Specify any assessment of risk of bias that may affect the cumulative evidence (e.g. publication bias, selective reporting within studies). | 2.5 |
| 16 | Describe methods of additional analyses (e.g., sensitivity or subgroup analyses, meta-regression), if done, indicating which were pre-specified. | 2.5 & 3.2 |
| **RESULTS** | | |
| 17 | Give numbers of studies screened, assessed for eligibility, and included in the review, with reasons for exclusions at each stage, ideally with a flow diagram. | Figure 1 |
| 18 | For each study, present data characteristics (e.g. study size, PICOS, follow-up period) and provide citations. | Table 2 |
| 19 | Present data on risk of bias of each study and, if available, any outcome level assessment (**see 12**). | Table 5 |
| 20 | For all outcomes considered (benefits or harms), present, for each study: (a) simple summary data for each intervention group (b) effect estimates and confidence intervals, ideally with a forest plot. | Figures 2, 4-6 |
| 21 | Present results of each meta-analysis done, including confidence intervals and measures of consistency. | 3.2 & 3.3 |
| 22 | Present results of any assessment of risk of bias across studies (**see 15**). | Table 5 |
| 23 | Give results of additional analyses, if done (e.g. sensitivity/subgroup analyses, meta-regression [**see 16**]). | Table 5 |

| **Item** | **Recommendation** | **Report Section** |
| --- | --- | --- |
| **DISCUSSION** | | |
| 24 | Summarize the main findings including the strength of evidence for each main outcome; consider their relevance to key groups (e.g., healthcare providers, users, and policy makers). | 4.1 & 4.4 |
| 25 | Discuss limitations at study and outcome level (e.g., risk of bias), and at review-level (e.g., incomplete retrieval of identified research, reporting bias). | 4.3 |
| 26 | Provide a general interpretation of the results in the context of other evidence, and future implications. | 4.2 |
| **FUNDING** | | |
| 27 | Describe sources of funding for the systematic review and other support (e.g., supply of data); role of funders for the systematic review. | Acknowledgements |

**Adapted From:** Moher D, Liberati A, Tetzlaff J, Altman DG, The PRISMA Group (2009). Preferred Reporting Items for Systematic Reviews and Meta-Analyses: The PRISMA Statement. PLoS Med, 6(7) https://www.bmj.com/content/bmj/339/bmj.b2535.full.pdf

**APPENDIX 3 | Details of the database search strategy, conducted on 14/11/18**

| **Database** | **Search Terms and Criteria** |
| --- | --- |
| **PubMed**  **(N = 504)** | **Search Terms:**  (((((((Hematological Malig*) OR Blood Cancer*) OR Leukemia) OR Lymphoma) OR Myeloma) OR Hodgkin*)) AND ((((((Refiner*) OR Petroleum) OR Petrochemical*) OR Oil Ind*) OR Gas Ind*) OR Chemical Ind*)  **Criteria:**  (a) All Years, (b) Species = Humans, (c) All Languages, (d) Availability = Full Text |
| **Web of Science (N = 452)** | **Search Terms:**  TS = (“Hematological Malig*” OR “Blood Cancer*” OR “Leukemia” OR “Lymphoma” OR “Myeloma” OR “Hodgkin*”) AND TS = ("Refiner*" OR "Petroleum" OR "Petrochemical*" OR "Oil Ind*" OR "Gas Ind*" OR "Chemical Ind*")  **Criteria:**  (a) All Years, (b) Only Research Articles, (c) All Languages |
| **Cochrane**  **(N = 69)** | **Search Terms:**  (“Hematological Malig*” OR “Blood Cancer*” OR “Leukemia” OR “Lymphoma” OR “Myeloma” OR “Hodgkin*”) AND ("Refiner*" OR "Petroleum" OR "Petrochemical*" OR "Oil Ind*" OR "Gas Ind*" OR "Chemical Ind*") |
| **ScienceDirect**  **(N = 359)** | **Search Terms (N = 17):**  (“Hematological Malignancy” OR “Hematological Malignancies”) AND ("Refinery" OR "Petroleum" OR "Petrochemical" OR "Oil" OR "Gas" OR "Chemical" OR “Industry”)  **Search Terms (N = 1):**  (“Blood Cancer” OR “Blood Cancers”) AND ("Refinery" OR "Petroleum" OR "Petrochemical" OR "Oil" OR "Gas" OR "Chemical" OR “Industry”)  **Search Terms (N = 243):**  (“Leukemia”) AND ("Refinery" OR "Petroleum" OR "Petrochemical" OR "Oil" OR "Gas" OR “Industry”)  **Search Terms (N = 106):**  (“Lymphoma”) AND ("Refinery" OR "Petroleum" OR "Petrochemical" OR "Oil" OR "Gas" OR “Industry”)  **Search Terms (N = 26):**  (“Myeloma”) AND ("Refinery" OR "Petroleum" OR "Petrochemical" OR "Oil" OR "Gas" OR “Industry”)  **Search Terms (N = 40):**  (“Hodgkin”) AND ("Refinery" OR "Petroleum" OR "Petrochemical" OR "Oil" OR "Gas" OR “Industry”)  **Criteria:**  (a) All Years, (b) Only Research Articles, (c) All Languages |

**APPENDIX 4 | Newcastle-Ottawa Quality Assessment Scale (Cohort Studies)**

| **NEWCASTLE-OTTAWA QUALITY ASSESSMENT (COHORT STUDIES)** | | |
| --- | --- | --- |
| **AUTHOR:** | *Axelsson et al. (2010)* | **COMMENTS** |
| **TITLE:** | *Cancer incidence in a petrochemical industry area in Sweden* |  |
| **SELECTION:** | | **★★★☆** |
| **1) Representativeness of the exposed cohort**  Truly representative (+1)  Somewhat representative [e.g. random sample of exposed persons] (+1)  Selected group of patients (0)  no description of the derivation of the cohort (0) | | *Page 4483:*  *The exposed cohort includes all persons residing within ‘high’ exposure zones in the Stenungsund municipality.* |
| **2) Selection of the non-exposed cohort**  Drawn from the same community as the exposed cohort (+1)  Drawn from a different source [e.g. outside of the study city or region] (0)  No description of the derivation of the non-exposed cohort (0) | | *Pages 4483 and 4485 (Table 5):*  *The non-exposed cohort is also from the Stenungsund municipality, offering a direct comparison.* |
| **3) Ascertainment of exposure ***  Geolocation obtained from a secure record [e.g. census or medical] (+1)  Geolocation obtained from a structured interview (+1)  Self-reported residency (0)  No description (0) | | *Page 4483 (Figure 1):*  *Cancer registry observations were linked to zones of ‘high’ (≤2km from a facility) and ‘low’ exposure to measured VOC concentrations.* |
| **4) Demonstration that outcome of interest was not present at start of study**  Yes (+1)  No (0) | |  |
| **COMPARABILITY:** | | **★☆** |
| **5) Comparability of the cohorts based on design or analysis [multiple answers]**  Study controls for age and gender (+1)  Study controls for any additional factors [e.g. socio-economic status] (+1) | | *Page 4485 (Table 4):*  *Standardised incident rates control for age and gender.* |
| **OUTCOME:** | | **★★☆** |
| **6) Assessment of outcome**  Independent blind assessment (+1)  Record linkage [e.g. Diagnosis obtained from a medical registry] (+1)  Self-reported (0)  No description (0) | | *Page 4483:*  *Residents linked to cancer registry records.* |
| **7) Was follow up long enough for outcomes to occur? ***  Yes, if the cohort is ≤ 15 years of age or the study duration is ≥ 10 years (+1)  No, if the duration of study is < 10 years (0) | | *Page 4485 (Table 4):*  *Cancer incidents were examined in the high/low exposure cohorts between 1994 and 2005.* |
| **8) Adequacy of follow up of cohorts**  Complete follow up - all subjects accounted for (+1)  Subjects lost to follow up unlikely to introduce bias: ≤ 20% lost, or description of those lost suggesting no different from those followed (+1)  Follow-up rate < 80% and no description of those lost (0)  No statement (0) | | *No information was provided on the number of residents that relocated into or out of the study areas.* |
| **OVERALL SCORE: ★ ★ ★ ★ ★ ★ ☆ ☆ ☆** | | |

**Adapted From:** <http://www.ohri.ca/programs/clinical_epidemiology/oxford.asp>

**APPENDIX 5 | Newcastle-Ottawa Quality Assessment Scale (Case-Control Studies)**

| **NEWCASTLE-OTTAWA QUALITY ASSESSMENT (CASE-CONTROL STUDIES)** | | |
| --- | --- | --- |
| **AUTHOR:** | *De Roos et al. (2010)* | **COMMENTS** |
| **TITLE:** | *Residential proximity to industrial facilities and risk of non-Hodgkin lymphoma (NHL)* |  |
| **SELECTION:** | | **★★★★** |
| **1)**  **Is the case definition adequate? ***  Yes, with independent validation (+1)  Yes, with record linkage or based on self-reports (0)  No description (0)  ** Cases are persons diagnosed with a haematological malignancy. These are compared to people with comparable lifestyles and socio-environmental exposures, who do not have a haematological malignancy (i.e. the control group).* | | *Page 71:*  *The US National Cancer Institute SEER database contains records of NHL incidence diagnosed by health care professionals, in accordance to the ICD-0 classification system. These diagnoses were also validated by interviews.* |
| **2)**  **Representativeness of the cases**  Includes all eligible or an obviously representative series of cases (+1)  Potential for selection biases or not stated (0) | | *Page 71:*  *Includes all cases of newly diagnosed NHL for persons age 20-74 without HIV, located within 4 US States from 1998-2000.* |
| **3)**  **Selection of Controls**  Community controls (+1)  Hospital controls (+1)  No description (0) | | *Page 71:*  *The controls were acquired from a health insurance provider, and matched to the cases by age, sex and race.* |
| **4)**  **Definition of Controls**  No history of disease (+1)  No description of source (0) | | *Page 73 (Table 1):*  *95% of cases and controls have no family history of NHL* |
| **COMPARABILITY:** | | **★★** |
| **5) Comparability of the cohorts based on design or analysis [multiple answers]**  Study controls for age and gender (+1)  Study controls for any additional factors [e.g. socio-economic status] (+1) | | *Page 73 (Table 1):*  *Controls for age, gender, ethnicity, BMI, smoking status, family history, education, etc.* |
| **EXPOSURE:** | | **★★☆** |
| **6) Ascertainment of exposure ***  Geolocation obtained from a secure record [e.g. census or medical] (+1)  Geolocation obtained from a blind structured interview (+1)  Geolocation obtained from unblinded interview (0)  Self-reported (0)  No description (0) | | *Page 71:*  *Current address obtained from SEER and Medicare registries. GPS coordinates validated during follow-up interview(s).* |
| **7)**  **Same method of ascertainment for cases and controls**  Yes (+1)  No (0) | | *Page 71 and 74:*  *Exposed persons were defined as living within 2 miles of a facility, which is refining or manufacturing petroleum products (SIC code 29)* |
| **8)**  **Non-Response rate**  Similar rate of response for both groups [within ± 10%] (+1)  Rate different and no designation (0)  No description (0) | | *Page 71:*  *Response rate for ‘Cases’ = 59%*  *Response rate for ‘Controls’ = 44%*  *Difference in response rates = 15%* |
| **OVERALL SCORE: ★ ★ ★ ★ ★ ★ ★ ★ ☆** | | |

**Adapted From:** <http://www.ohri.ca/programs/clinical_epidemiology/oxford.asp>

**APPENDIX 6 | High resolution dasymetric modelled population counts of Louisiana, aggregated into 5x5km grids (Source:** **Dmowska & Stepinski 2014)**


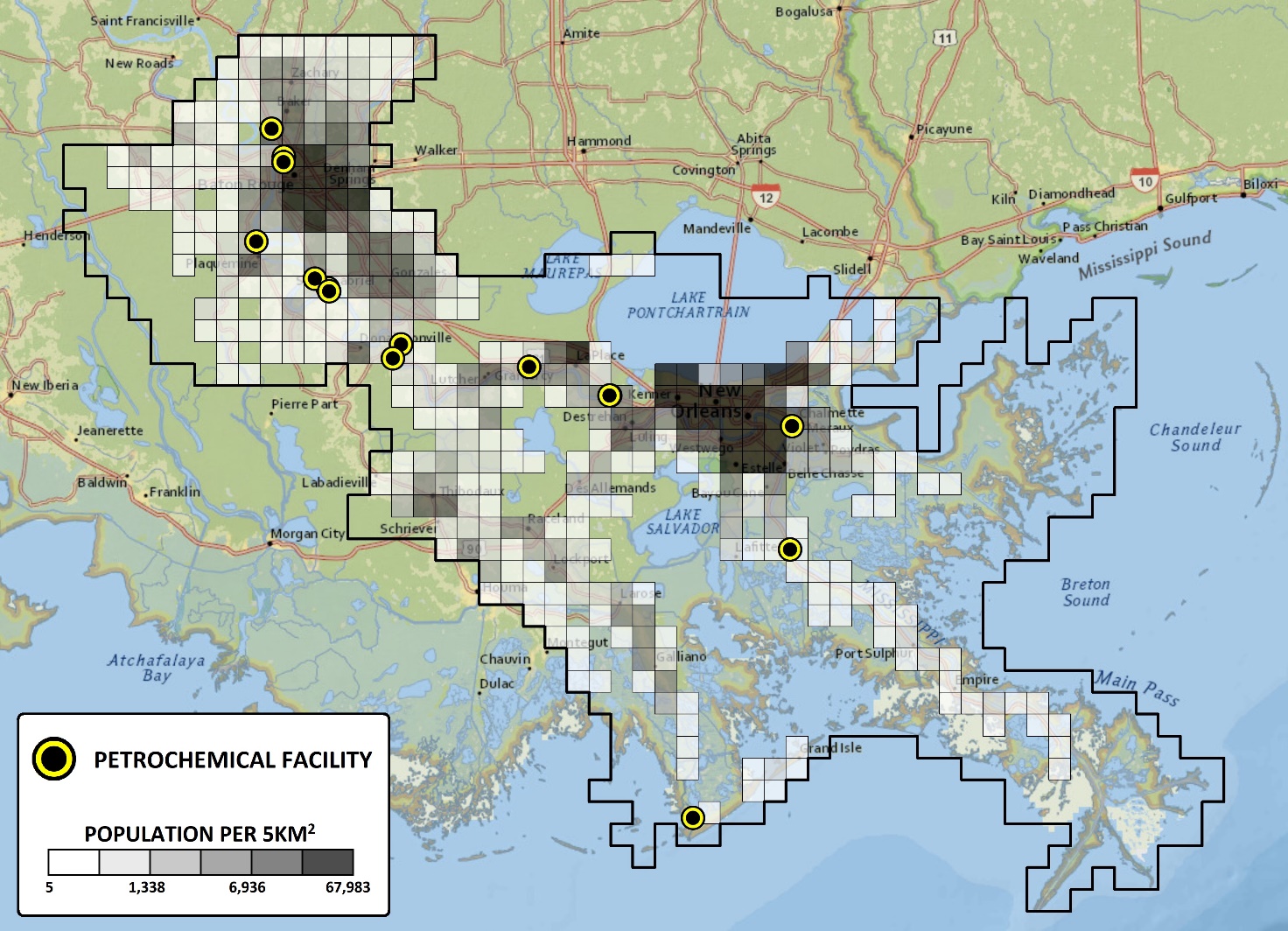


**APPENDIX 7 | Funnel plots for the association between residential exposure to petrochemical activity and the relative risk (RR) of Non-Hodgkin’s Lymphoma, Hodgkin’s Lymphoma and Multiple Myeloma incidence**


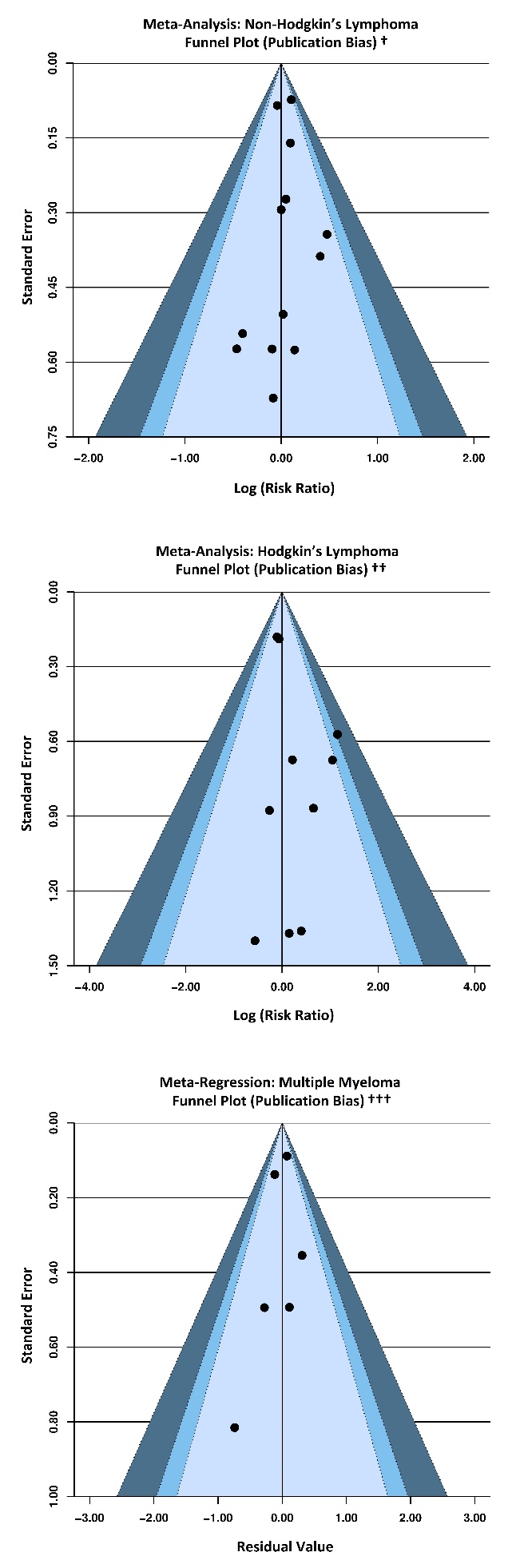


**⚫ Observed study estimates**

**⭘ Imputed study estimates from the “trim and fill” sensitivity analysis**

**† Egger’s regression test p-value = 0.85 †† Egger’s regression test p-value = 0.16 ††† Egger’s regression test p-value = 0.19**
